# Supplementary material for: A de novo evolved gene contributes to rice grain shape difference between indica and japonica
Source: Nat Commun. 2023 Sep 22;14:5906. doi: 10.1038/s41467-023-41669-w (PMC10516980; doi:10.1038/s41467-023-41669-w)
Supplement: Supplementary file 1 — Supplementary Information [file 41467_2023_41669_MOESM1_ESM.pdf]

A *de novo* evolved gene contributes to rice grain shape difference between *indica* and *japonica*

Chen *et al.*

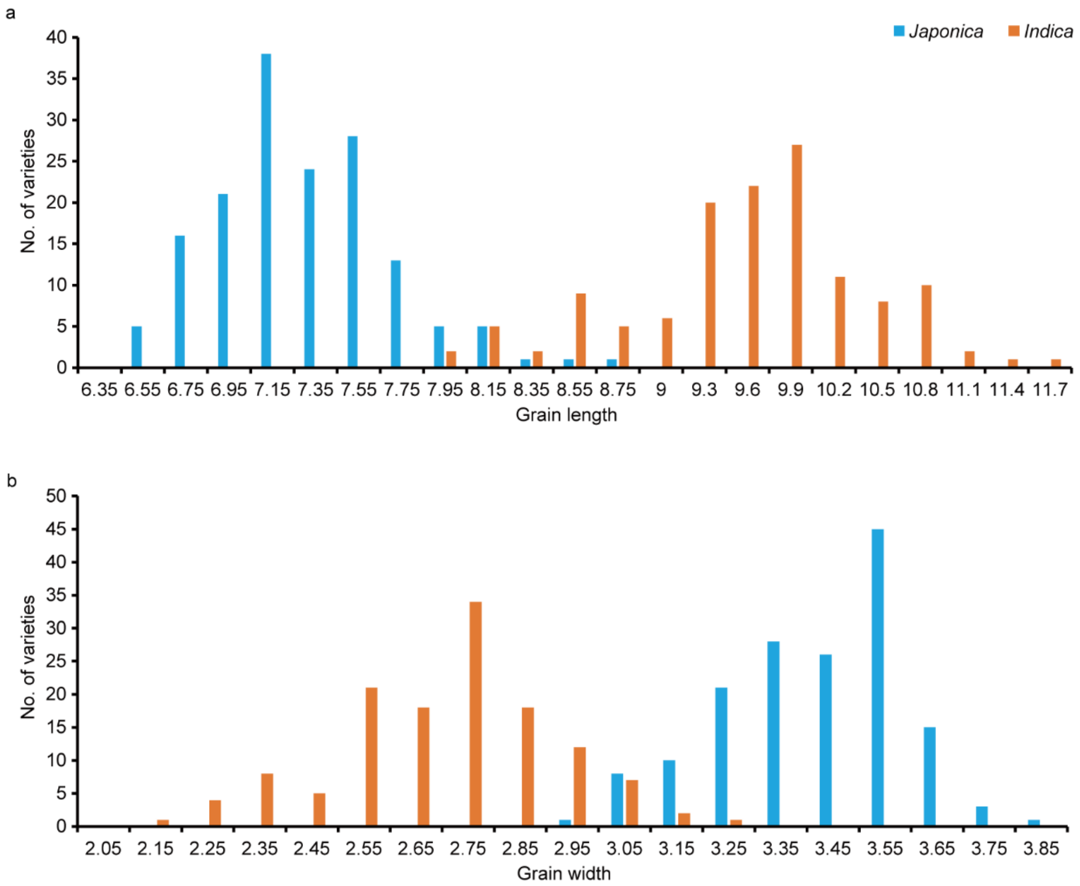

Supplementary Fig. 1. Grain shape variation among 131 *indica* and 158 *japonica* varieties. Frequency distributions of grain length (a) and grain width (b). Source data are provided as a Source Data file.

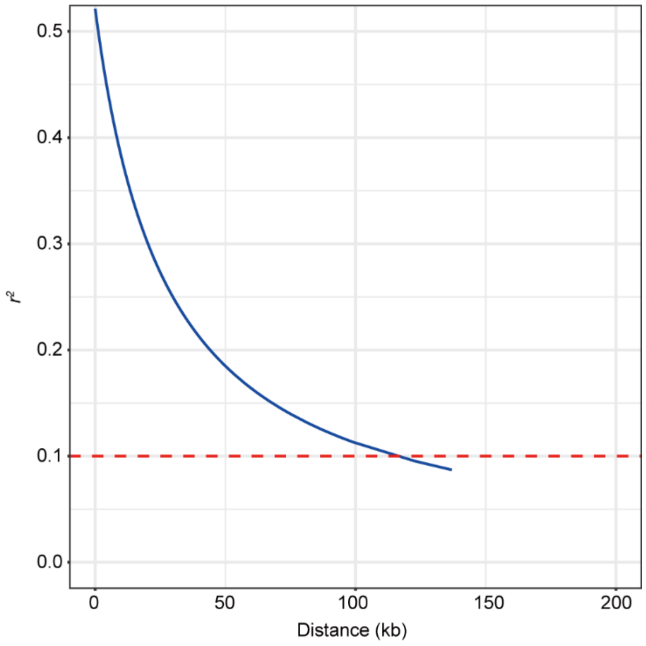

Supplementary Fig. 2. Genome-wide average linkage disequilibrium (LD) decay. The decay of LD with physical distance between SNPs occurred at about 110 kb (r^2 = 0.1).

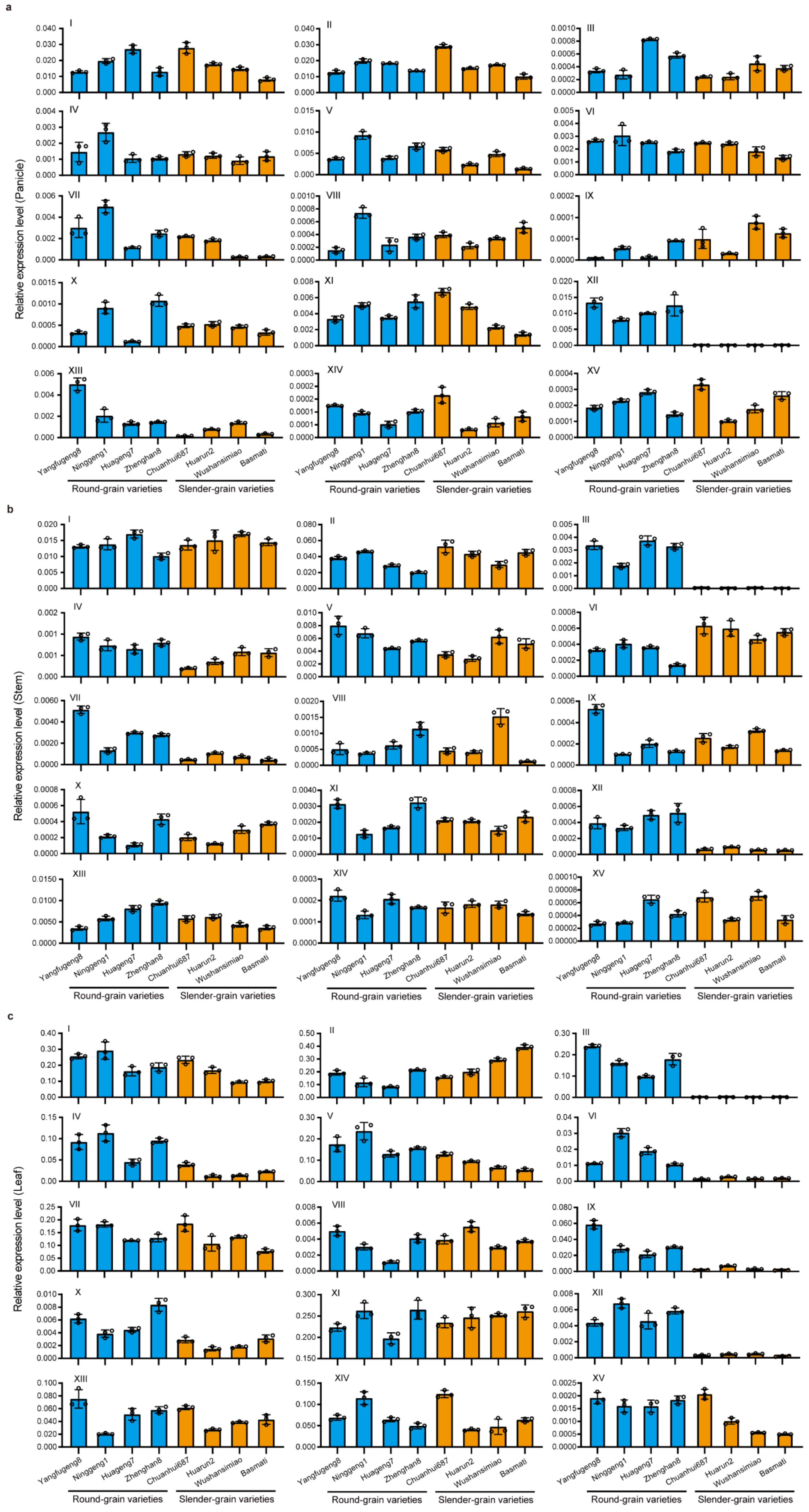

Supplementary Fig. 3. Expression levels of 15 candidate genes in eight rice varieties. Relative expression levels of 15 candidate genes in the young panicle (a), stem (b) and leaf (c) of four representative slender-grain and round-grain varieties, respectively. *OsActin* was used as a control. Data shown are means ± SD (n = 3 biological replicates). Source data are provided as a Source Data file.

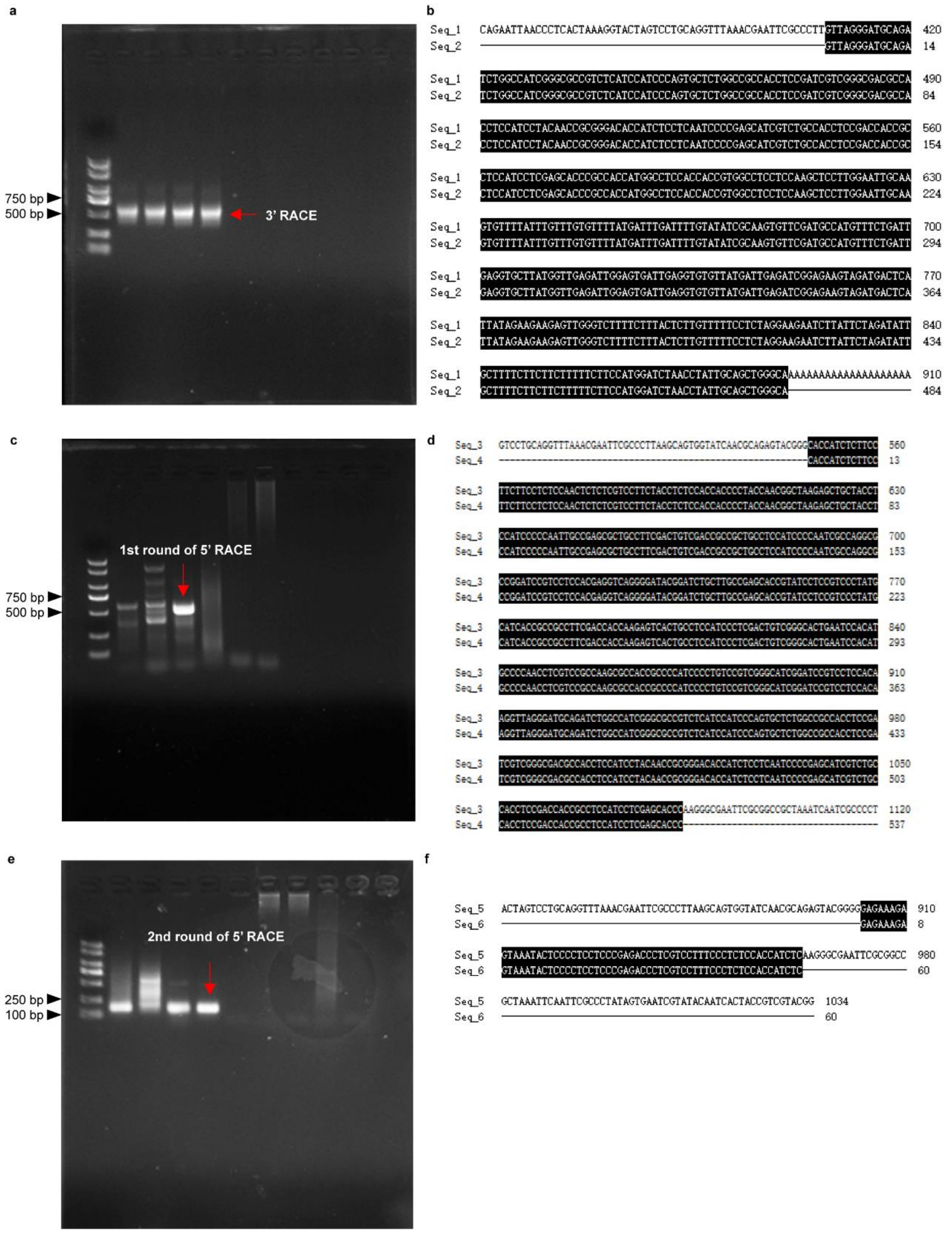

Supplementary Fig. 4. The full-length *GSE9* gene confirmed by rapid-amplification of cDNA ends (RACE). (a-b) Uncropped gel images for 3' RACE (a) and the alignment of its sequencing results with the annotated sequence (b). (c-d) Uncropped gel images for the first round of 5' RACE (c) and the alignment of its sequencing results with the annotated sequence (d). (e-f) Uncropped gel images for the second round of 5' RACE (e) and the alignment of its sequencing results with the annotated sequence (f). The red arrow indicates the specific products of RACE that are further used for sequencing.

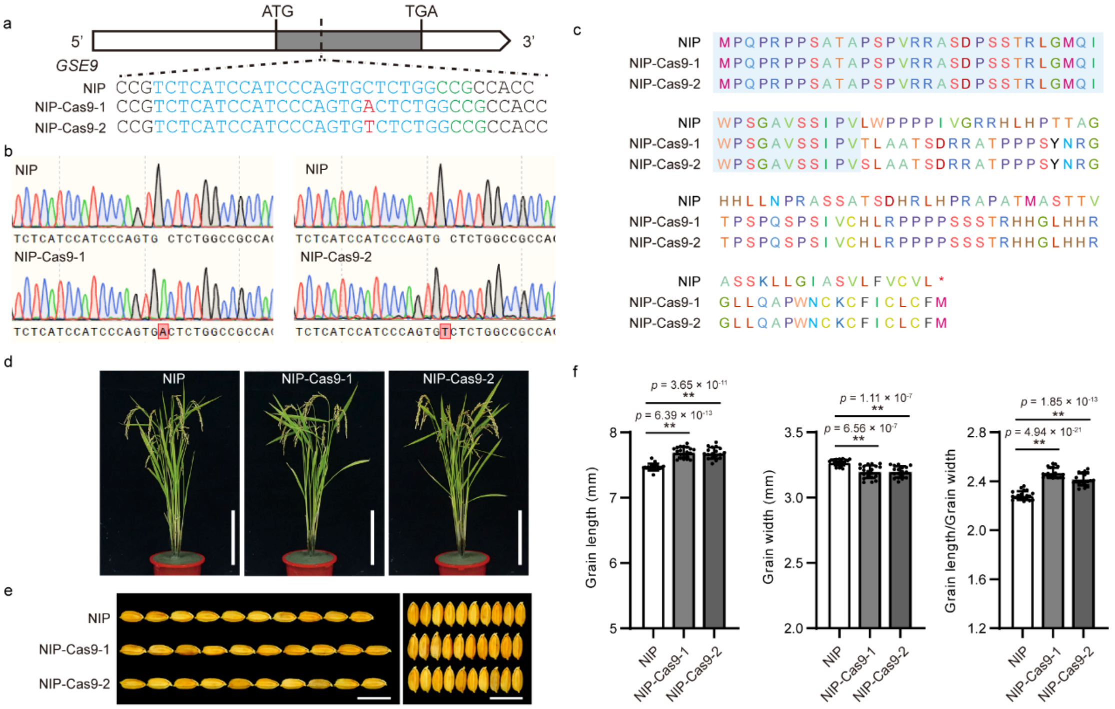

Supplementary Fig. 5. Identification and phenotypic characterization of the *GSE9* knockout mutants in Nipponbare background. (a-c) Identification of *GSE9* knockout mutants generated by the CRISPR/Cas9 system in Nipponbare (NIP) background. (a) Targeted mutagenesis of *GSE9*. The target mutated sites are indicated on the gene structure of *GSE9*. Grey box indicates the single exon of *GSE9* gene. (b) Mutation events were confirmed by sequencing. The target mutated sites are marked with red box. (c) Comparison of amino acid sequences between NIP and *GSE9* knockout mutants. Red asterisk indicates the termination codon. (d-f) Phenotypic identification of *GSE9* knockout mutants in NIP background. (d) Plant morphology of NIP and *GSE9* knockout mutants at the mature stage. Scale bar, 20 cm. (e) Comparison of grain shape between NIP and *GSE9* knockout mutants. Scale bar, 1 cm. (f) Statistical analysis of grain length, grain width, and grain length/width ratio between NIP and *GSE9* knockout mutants. Data show means ± SD (n = 20/20/18, 20/20/18, 20/20/18 biological replicates). Statistical analysis was performed by two-tailed Student's t-test (**, $p < 0.01$). Source data are provided as a Source Data file.

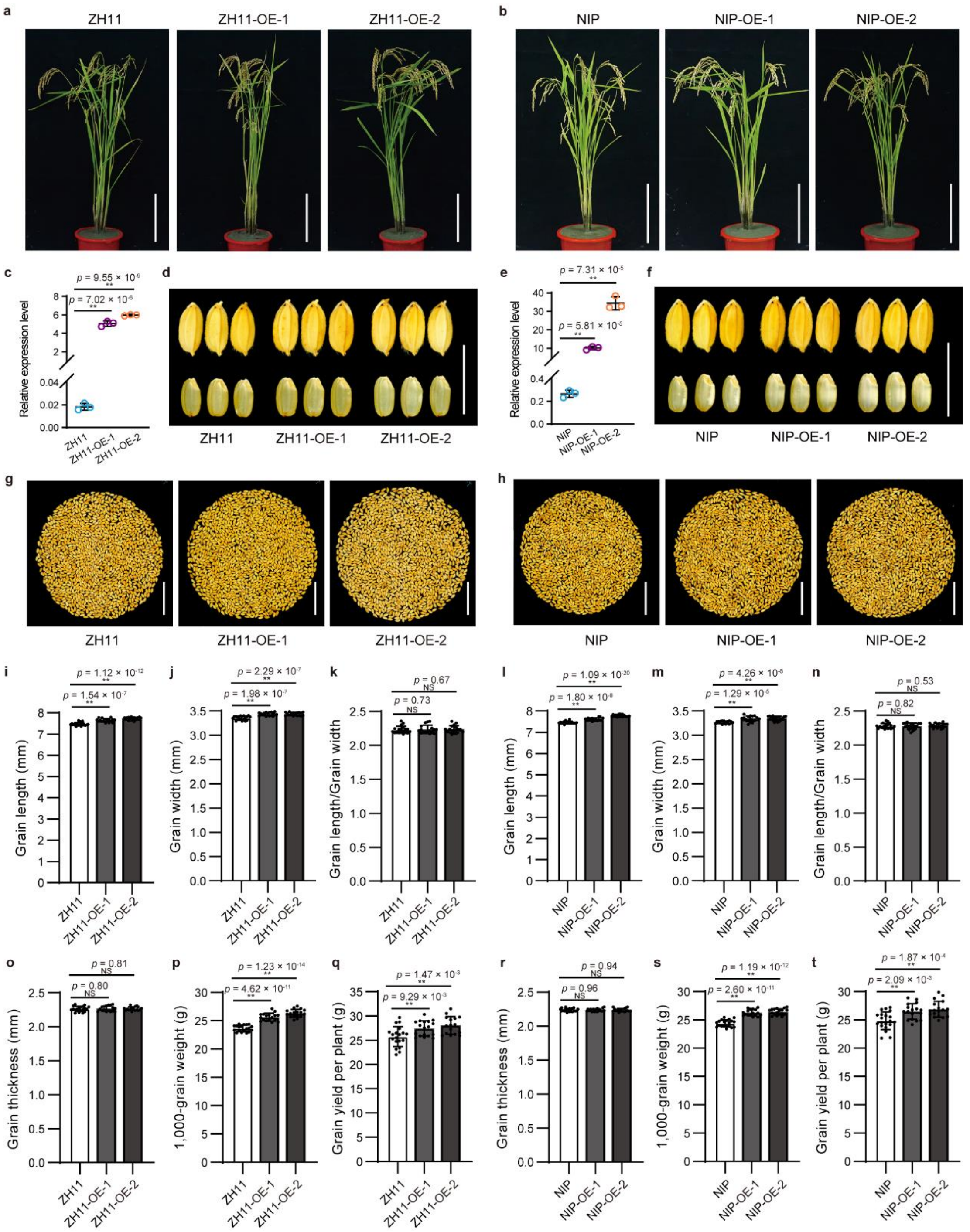

Supplementary Fig. 6. Phenotypic identification of *GSE9* overexpressing lines in Zhonghua11 and Nipponbare backgrounds. (a-b) Plant morphology of the wild-type and *GSE9* overexpressing lines at the mature stage. Scale bar, 20 cm. (c) *GSE9* transcript levels in ZH11 and ZH11-OE lines determined by qRT-PCR analysis. (d) Comparison of the grain shape between ZH11 and ZH11-OE lines. (e) *GSE9* transcript levels in NIP and NIP-OE lines determined by qRT-PCR analysis. The expression level of *OsActin* was used as a control. (f) Comparison of grain shape between the NIP and NIP-OE lines. (g-h) Comparison of grains per plant between WT and *GSE9*-OE lines. Scale bar, 5 cm. (i-t) Comparison of grain length (i, l), grain width (j, m), grain length/grain width ratio (k, n), grain thickness (o, r), 1,000-grain weight (p, s) and grain yield per plant (q, t) between WT and *GSE9*-OE lines. Data show means ± SD (n = 3 biological replicates in c and e; n = 19/17/17 biological replicates in i-k, o-p; n = 19/18/16 biological replicates in q; n = 20/17/18 biological replicates in l-n, r-t). Statistical analysis was performed by two-tailed Student's t-test (**, $p < 0.01$; NS, not significant). Source data are provided as a Source Data file.

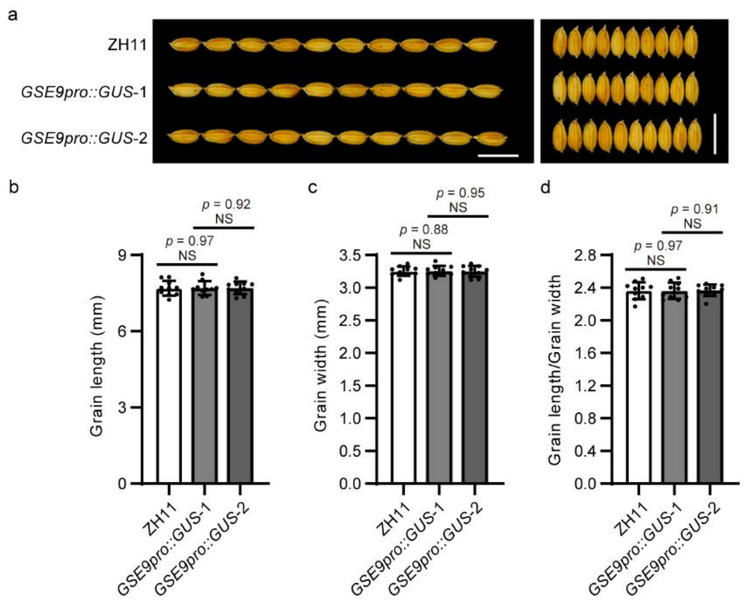

Supplementary Fig. 7. Phenotypic identification of *GSE9pro::GUS* transgenic plants in the *japonica* variety Zhonghua11 background. (a) Morphology of grain shape in the wild-type ZH11 and *GSE9pro::GUS* transgenic plants. Scale bar, 1 cm. (b-d) Comparison of grain length (b), grain width (c) and grain length/width ratio (c) between ZH11 and *GSE9pro::GUS* transgenic lines. Data show means ± SD (n = 10 biological replicates). Statistical analysis was performed by two-tailed Student's t-test (NS, not significant). Source data are provided as a Source Data file.

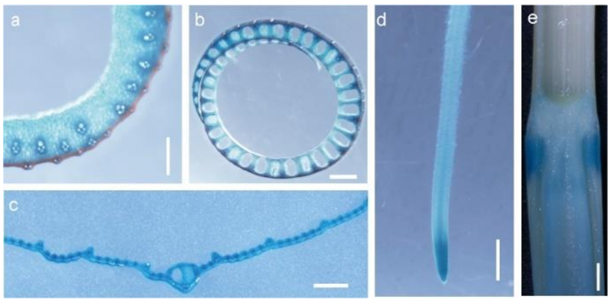

Supplementary Fig. 8. GUS staining analysis of various tissues in *GSE9pro::GUS* transgenic plants. Histochemical GUS staining in stem (a), sheath (b), leaf (c), root (d), and node (e). Scale bar, 500 μm in (a), 1 mm in (b, d), 2 mm in (c, e). At least three independent replicates were performed and a representative result is shown.

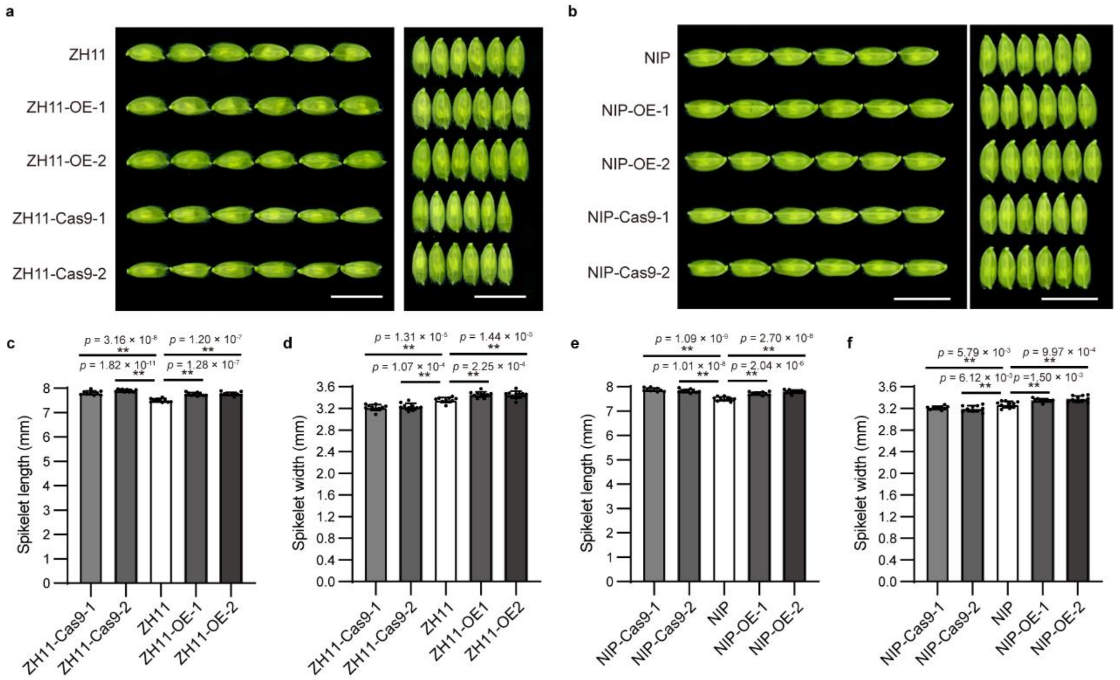

Supplementary Fig. 9. Phenotypic identification of spikelet hulls between wild-type and *GSE9* transgenic lines in Zhonghua11 and Nipponbare backgrounds. (a-b) Morphology of spikelet hulls. Scale bar, 1 cm. (c-f) Comparison of spikelet length and width between WT and *GSE9* transgenic lines. Data show means ± SD (n = 10 biological replicates). Statistical analysis was performed by two-tailed Student's t-test (**, $p < 0.01$). Source data are provided as a Source Data file.

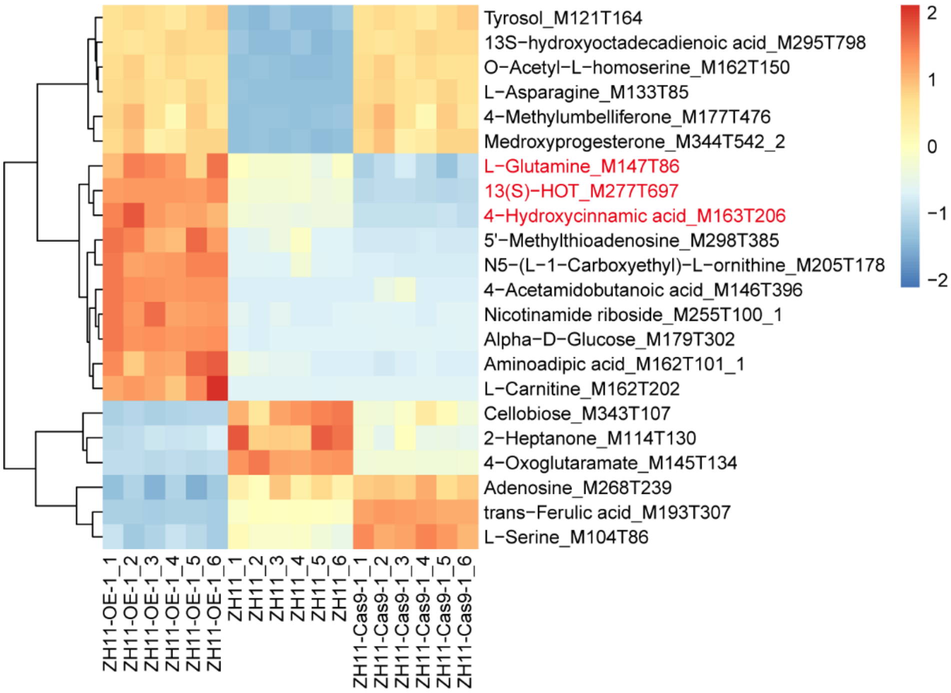

Supplementary Fig. 10. Heatmap showing the relative levels of all differential metabolites in young panicles under ZH11-OE-1 *vs* ZH11 and ZH11-Cas9-1 *vs* ZH11 comparisons. Metabolites were extracted from young panicle samples with six biological replicates from ZH11, ZH11-OE-1 and ZH11-Cas9-1. The metabolite profiling was completed using LC-MS. The orthogonal partial least squares discriminant analysis (OPLS-DA) was used to calculate the Variable Importance for the Projection (VIP). VIP >1 and P value < 0.05 were used as the criteria to identify differential metabolites among different comparisons. Red fonts indicate the three overlapped metabolites between ZH11-OE-1 *vs* ZH11 and ZH11-Cas9-1 *vs* ZH11 comparisons. Source data are provided as a Source Data file.

a

Description	Scientific Name	Max Score	Total Score	Query Cover	E value	Per. Ident	Acc. Len	Accession
Oryza sativa Japonica Group cDNA clone:J023124E17, full insert sequence	Oryza sativa Japonica Group	206	206	100%	9e-64	100.00%	928	AK072475.1
Oryza sativa Japonica Group genomic DNA, chromosome 9, BAC clone:OSJNBa0038I09	Oryza sativa Japonica Group	206	206	100%	8e-59	100.00%	153520	AP005727.3
Oryza sativa Japonica Group genomic DNA, chromosome 9, BAC clone:OSJNBa0011D16	Oryza sativa Japonica Group	206	206	100%	8e-59	100.00%	177329	AP005892.3
Oryza sativa Indica Group cultivar Zhenshan 97 chromosome 9	Oryza sativa Indica Group	206	206	100%	8e-59	100.00%	22989350	CP056060.1
Oryza sativa Japonica Group DNA, chromosome 9, cultivar: Nipponbare, complete sequence	Oryza sativa Japonica Group	206	206	100%	8e-59	100.00%	23012720	AP014965.1
Oryza sativa Indica Group cultivar RP Bio-226 chromosome 9 sequence	Oryza sativa Indica Group	197	197	100%	4e-56	97.22%	20520899	CP012617.1
Oryza sativa Indica Group cultivar Shuhui498 chromosome 9 sequence	Oryza sativa Indica Group	197	197	100%	4e-56	97.22%	24760661	CP018165.1
Oryza sativa Indica Group cultivar Minghui 63 chromosome 9	Oryza sativa Indica Group	197	197	100%	4e-56	97.22%	24892599	CP054684.1

b

Organism	Blast Name	Score	Number of Hits	Description
Oryza sativa	monocots		8	
. Oryza sativa Japonica Group	monocots	206	4	Oryza sativa Japonica Group hits
. Oryza sativa Indica Group	monocots	206	4	Oryza sativa Indica Group hits

c

Analysis	Jobs
BLASTN	Job 1: BLASTN against Oryza punctata Oryza_punctata_v1.2 (Genomic sequence) Done: No hits found
	Job 2: BLASTN against Oryza barthii O.barthii_v1 (Genomic sequence) Done: 1 hit found [View results]
	Job 3: BLASTN against Oryza meridionalis Oryza_meridionalis_v1.3 (Genomic sequence) Done: No hits found
	Job 4: BLASTN against Oryza rufipogon OR_W1943 (Genomic sequence) Done: 1 hit found [View results]
	Job 5: BLASTN against Oryza brachyantha Oryza_brachyantha.v1.4b (Genomic sequence) Done: No hits found
	Job 6: BLASTN against Oryza glumipatula Oryza_glumaepatula_v1.5 (Genomic sequence) Done: 1 hit found [View results]
	Job 7: BLASTN against Oryza longistaminata O_longistaminata_v1.0 (Genomic sequence) Done: 1 hit found [View results]

Supplementary Fig. 11. BLAST result of the GSE9 sequences. (a) TBLASTN result of NCBI nucleotide nonredundant database (nt) using the GSE9 protein sequence (JGI ID: LOC_Os09g06719) from *Oryza sativa japonica* (Nipponbare). *E*-value cutoff = 1*e*-5. (b) Taxonomy distribution of hits generated from TBLASTN searches in (a). Hits were found only from *O. sativa japonica* and *O. sativa indica*. (c) BLASTN result of Gramene database using the coding sequence of *GSE9* as query (*E*-value cutoff = 1*e*-50). Hits were found from *O. rufipogon*, *O. barthii*, *O. glumaepatula*, and *O. longistaminata*, while no hits were found from *O. meridionalis*, *O. punctata*, and *O. brachyantha*.

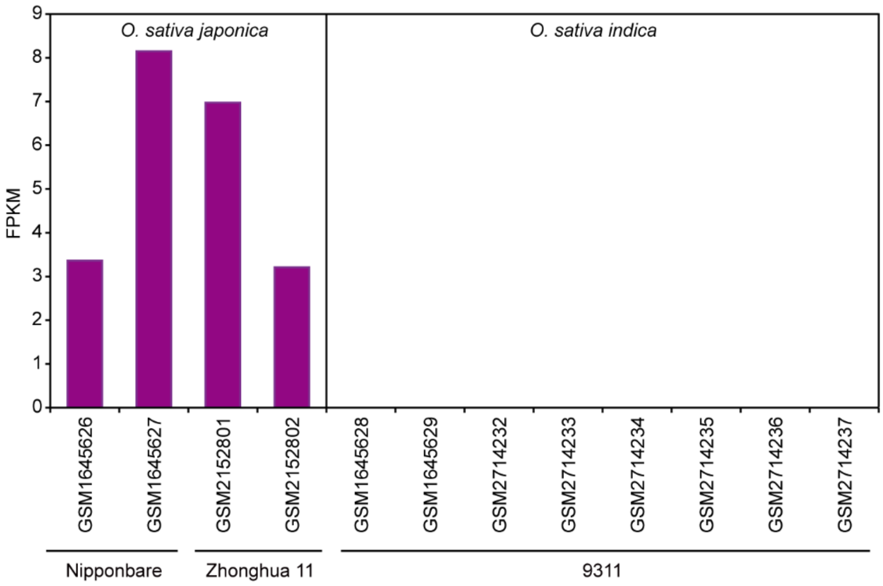

Supplementary Fig. 12. Transcriptional evidence for *GSE9* in *japonica*. RNA sequencing (RNA-Seq) data from the NCBI GEO DataSets for the transcriptional evidence of *GSE9* in *O. sativa*. FPKM values on the vertical axis indicate the expression level of *GSE9* in various NCBI GEO datasets on the horizontal axis. Source data are provided as a Source Data file.

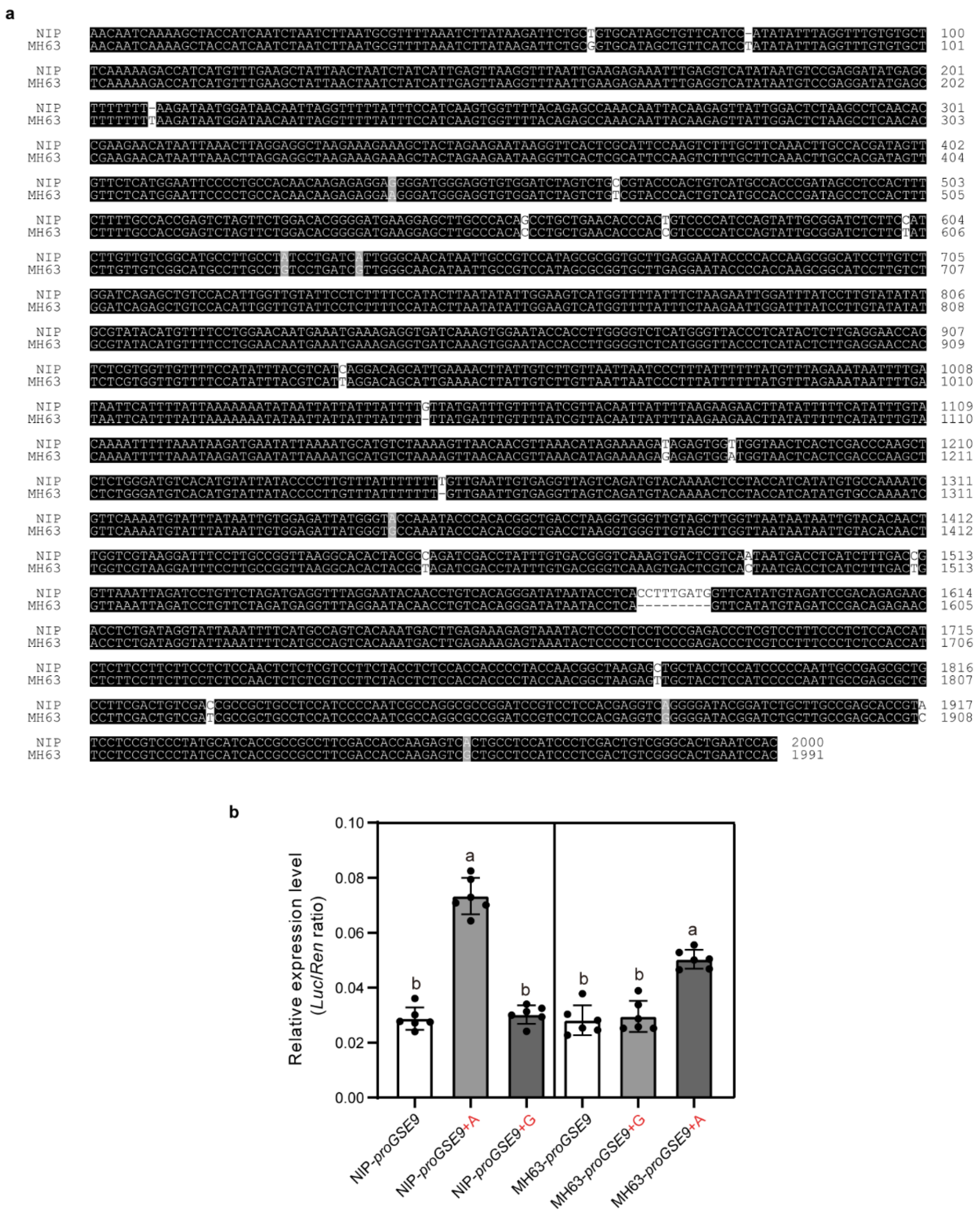

Supplementary Fig. 13. Comparison of the sequence and transcription activity of the promoter regions of *GSE9* between *japonica* and *indica*. (a) Alignment of the approximately 2-kb promoter region of *GSE9* between the *japonica* variety Nipponbare (NIP) and the *indica* variety Minghui 63 (MH63). (b) Transcription activity assays of the sequence of the promoter region, *proGSE9*+A and *proGSE9*+G sequence of *GSE9* locus in NIP and MH63. Data show means ± SD (n = 6 biological replicates). Different letters indicate statistically significant differences at $p < 0.05$ by one-way ANOVA test. Source data are provided as a Source Data file.

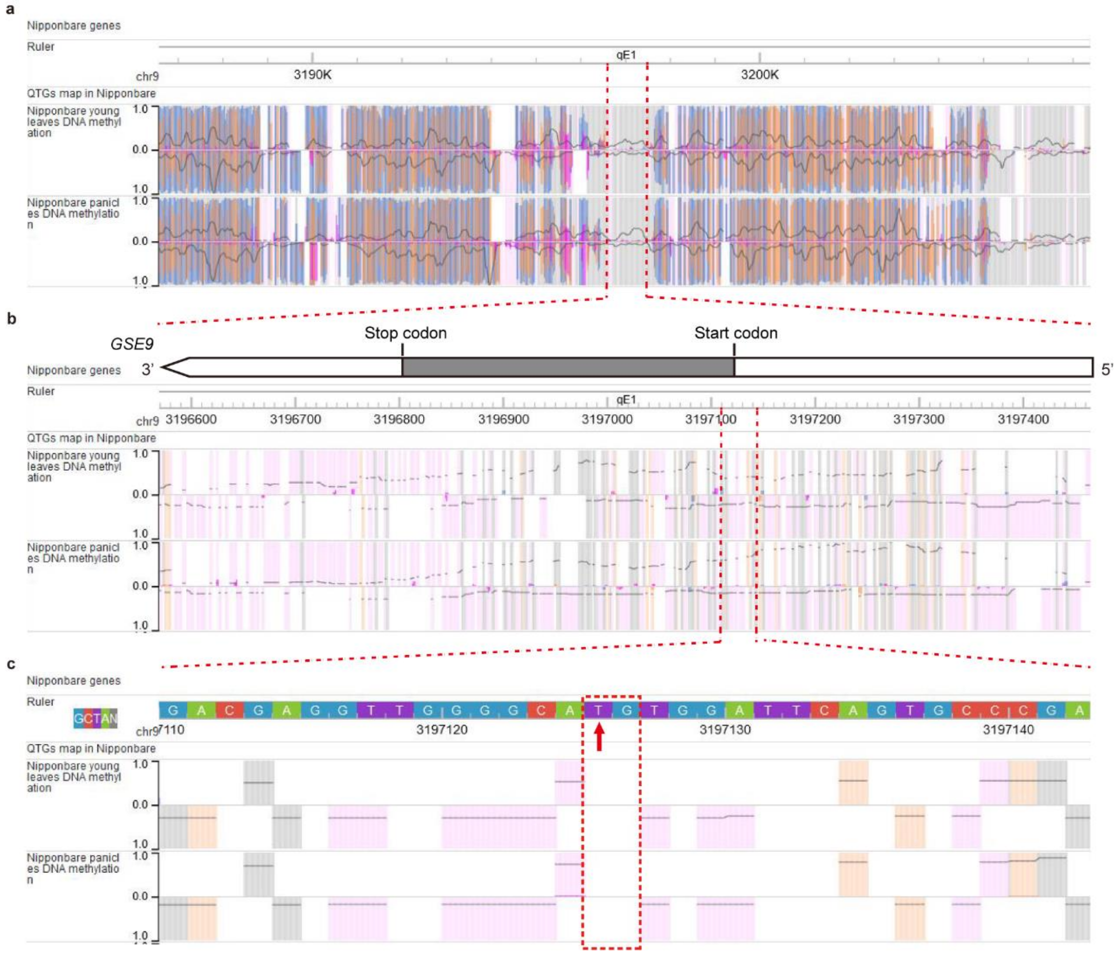

Supplementary Fig. 14. Three types of DNA methylation levels of *GSE9* locus and its flanking regions in young leaves and panicles of the *japonica* variety Nipponbare (NIP). (a) DNA methylation levels of *GSE9* locus and its flanking regions in NIP. Red dotted lines indicate the *GSE9* locus. (b) DNA methylation levels of the genomic region of *GSE9* in NIP. Grey box indicates the single exon of *GSE9* gene, while white boxes indicate its 5'-UTR and 3'-UTR, respectively. Red dotted lines indicate the sites surrounding the start codon of *GSE9*. (c) DNA methylation levels of the sites surrounding the start codon of *GSE9* in NIP. Red arrow indicates the nucleotide A at the start codon of *GSE9*. Red box shows no DNA methylation level in the start codon site of *GSE9*. The data are available from rice methylation database RiceENCODE. Bright blue, orange and magenta boxes indicate the CG, CHG and CHH methylation levels, respectively. Light blue, orange and magenta boxes indicate no CG, CHG and CHH methylation levels.

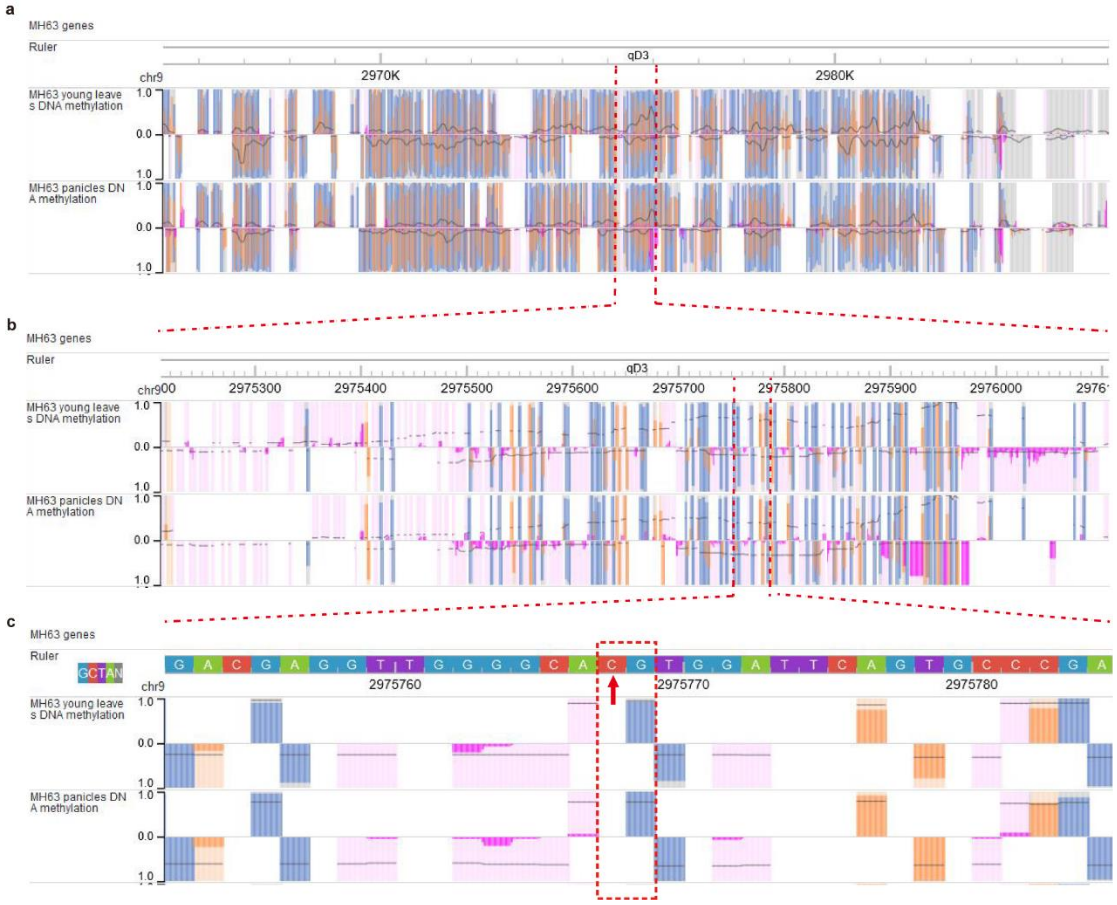

Supplementary Fig. 15. Three types of DNA methylation levels of homologous sequences with *GSE9* and its flanking regions in young leaves and panicles of the *indica* variety Minghui 63 (MH63). (a) DNA methylation levels of homologous sequences with *GSE9* and its flanking regions in MH63. Red dotted lines indicate the homologous sequences with *GSE9* genomic region. (b) DNA methylation levels of the homologous sequences with NIP *GSE9* genomic region in MH63. Red dotted lines indicate the homologous sites surrounding the start codon of NIP *GSE9* in MH63. (c) DNA methylation levels of the homologous sites surrounding the start codon of NIP *GSE9* in MH63. Red arrow indicates the G variation at the start codon of *GSE9* in MH63. Red box shows high CG methylation level in the start codon site of *GSE9* in MH63. The data are available from rice methylation database RiceENCODE. Bright blue, orange and magenta boxes indicate the CG, CHG and CHH methylation levels, respectively. Light blue, orange and magenta boxes indicate no CG, CHG and CHH methylation levels.

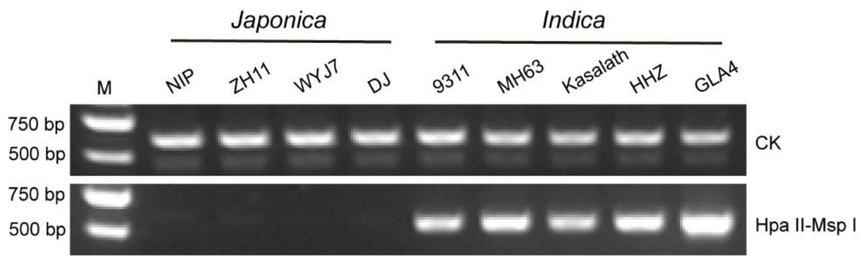

Supplementary Fig. 16. HpaII/MspI digestion-based PCR assay showing DNA methylation levels in the promoter region of *GSE9* in *japonica* and *indica*. The sampled *japonica* varieties include Nipponbare (NIP), Zhonghua11 (ZH11), Wuyunjing7 (WYJ7) and Dongjing (DJ). The sampled *indica* varieties include 9311, Minghui63 (MH63), Kasalath, Huanghuazhan (HHZ) and Guangluai4 (GLA4). The *GSE9* region cannot be digested in all detected *indica* varieties, but was digested in all sampled *japonica* varieties, suggestive of a higher level of DNA methylation in *indica*. M, molecular weight standard. Source data are provided as a Source Data file.

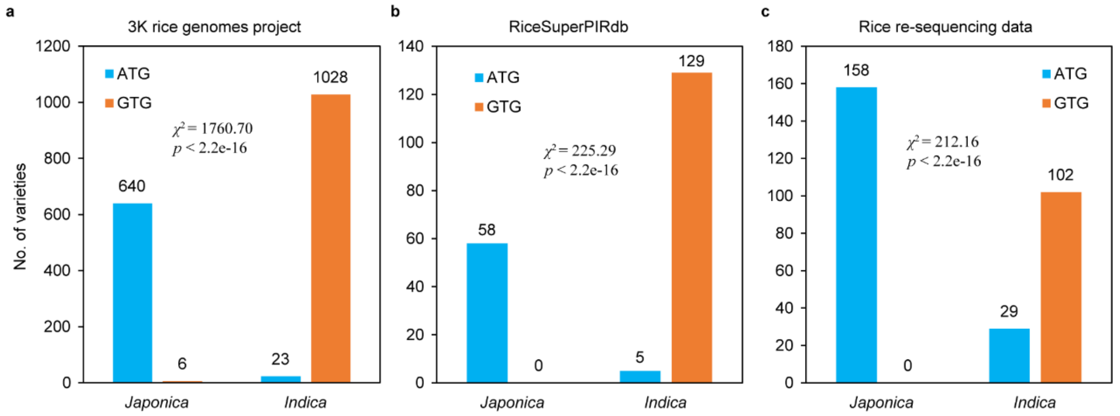

Supplementary Fig. 17. The distribution of A/G variation is unbalanced at the start codon site of *GSE9* between *japonica* and *indica* varieties. The distribution of A/G variation at the start codon site of *GSE9* in 1,697 rice varieties from 3K rice genomes project (a), 192 rice varieties from the Rice Super Pan-genome Information Resource Database (RiceSuperPIRdb) (b), and our re-sequencing 289 rice varieties (c).

Statistical analysis was performed by the chi-square (χ^2) test.

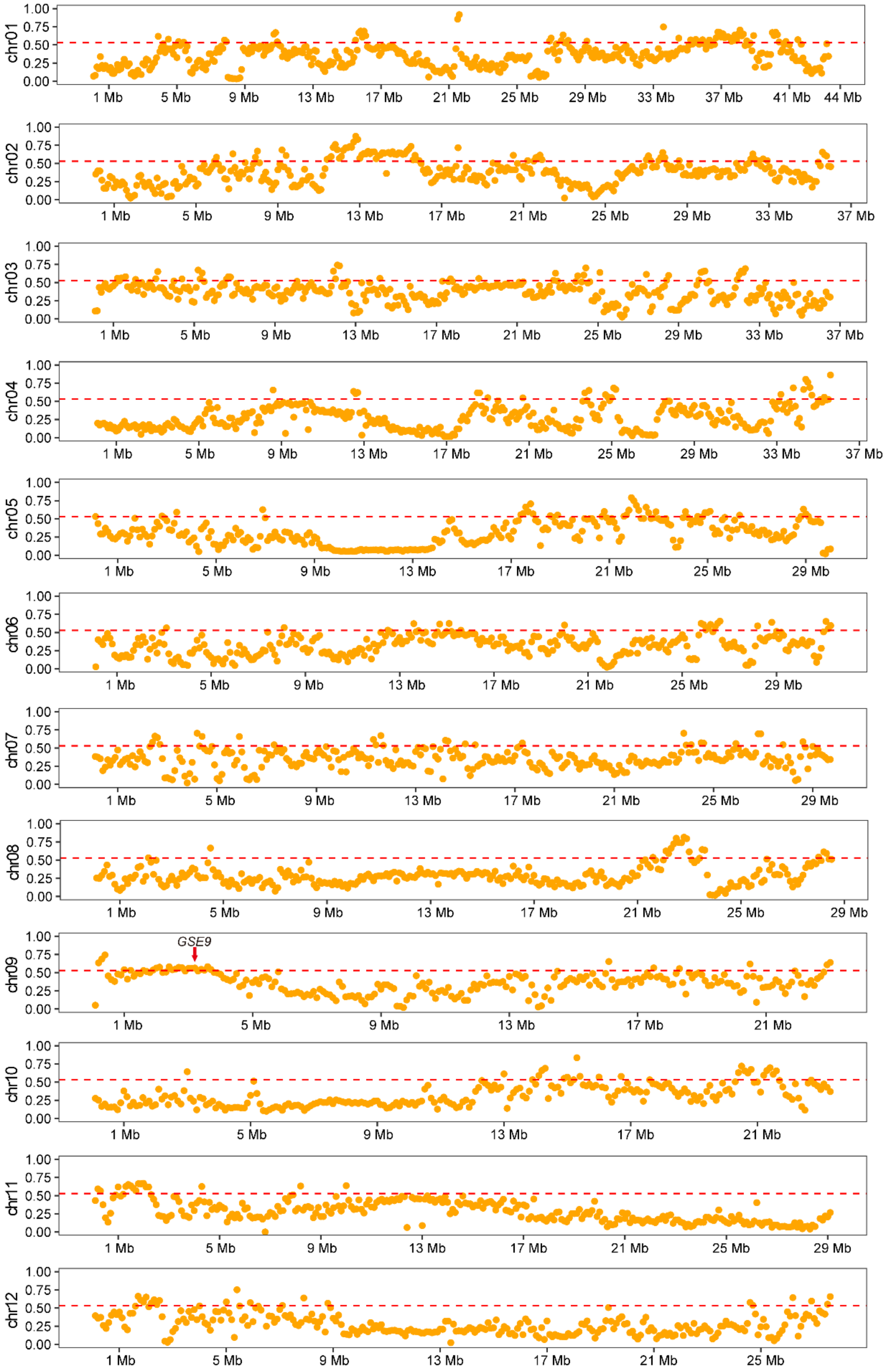

Supplementary Fig. 18. Plots of the population-differentiation statistic (F_{ST}) between *indica* and *japonica* across 12 rice chromosomes. The F_{ST} was estimated in each 100-kb window and the horizontal line indicates the threshold value of top 10% of F_{ST} at the whole-genome level between *indica* and *japonica*. Source data are provided as a Source Data file.

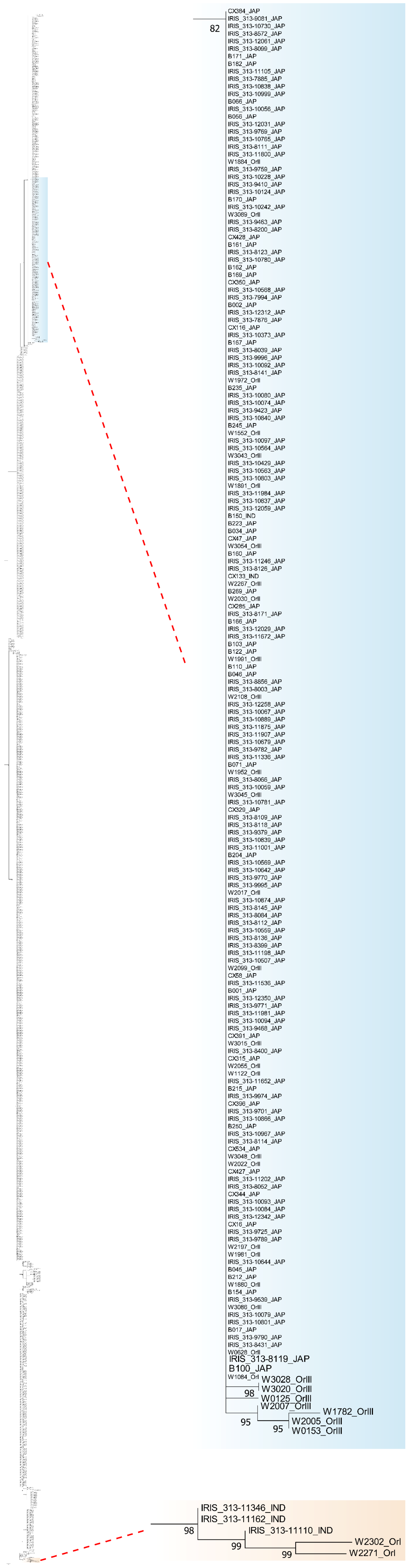

Supplementary Fig. 19. A traditional rectangular phylogenetic tree with bootstrap supporting values generated from the full-length cDNA sequence of *GSE9* in both cultivated rice and various groups of common wild rice varieties. The right-hand images show magnified views of the left boxed region.

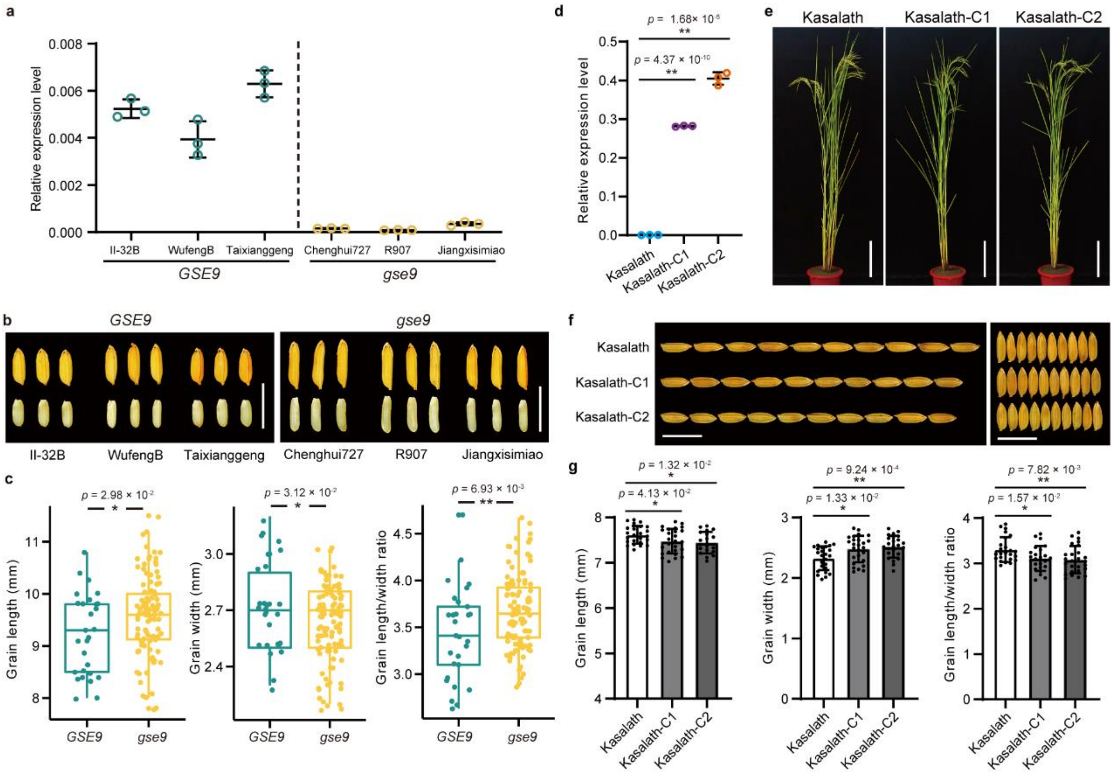

Supplementary Fig. 20. Natural variation and introgression of *GSE9* gene have effects on grain shape of rice. (a) Expression levels of *GSE9* gene in *GSE9* and *gse9* types of selected *indica* varieties determined by qRT-PCR analysis. (b) The phenotype of grain shape in *GSE9* and *gse9* types of the selected *indica* varieties. (c) Comparison of grain length, grain width, and grain length/width ratios between 29 *GSE9* and 102 *gse9* types of the *indica* varieties. In each box plot of c, the center line indicates the median, the edges of the box represent the first and third quartiles, and the whiskers extend to span a 1.5 interquartile range from the edges. (d) qRT-PCR analysis of the *GSE9* transcript levels in the Kasalath and *GSE9*-complementary lines. (e) Plant morphology of Kasalath and *GSE9*-complementary lines at the mature stage. (f) Comparison of grain shape between Kasalath and *GSE9*-complementary lines. The awn of grains was removed. (g) Statistical analysis of grain length, grain width, and grain length/width ratio between Kasalath and *GSE9*-complementary lines. The expression level of *OsActin* was used as a control. Data show means ± SD (n = 3 biological replicates in a and d; n = 24/26/19, 25/6/25, 24/24/26 biological replicates in g). Scale bar, 1 cm in b and f; 20 cm in e. Statistical analysis was performed by two-tailed Student's t-test (**, $p < 0.01$; *, $p < 0.05$). Source data are provided as a Source Data file.

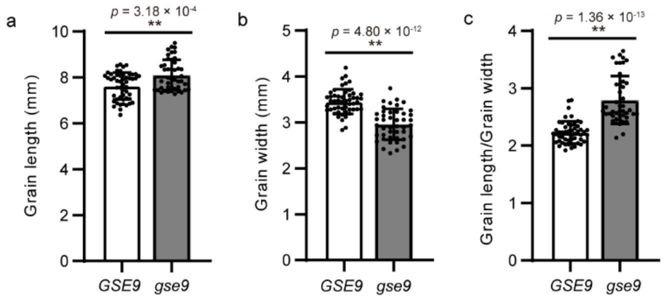

Supplementary Fig. 21. Comparison of grain shape between *Oryza rufipogon* with the *GSE9* type and *gse9* type. Statistical analysis of grain length (a), grain width (b), and grain length/width ratio (c). Data show means ± SD (n = 51 in the *GSE9* type; n = 44 in the *gse9* type). Statistical analysis was performed by two-tailed Student's t-test (**, $p < 0.01$). Source data are provided as a Source Data file.

Supplementary Table 1. Annotation of candidate genes anchored by the leading SNP on Chromosome 9 (±110 kb range) associated with the grain shape.

Locus	ID	Location	Description
LOC_Os09g06464	I	chr09: 3048020..3064571	CCT/B-box zinc finger protein,
LOC_Os09g06499	II	chr09: 3073884..3092870	sulfate transporter 4 (SULTR4)
LOC_Os09g06520	III	chr09: 3096690..3100658	expressed protein
LOC_Os09g06530	IV	chr09: 3104108..3105511	expressed protein
LOC_Os09g06560	V	chr09: 3118920..3125244	chromosome transmission fidelity protein 4 (WDHD1, CTF4)
LOC_Os09g06570	VI	chr09: 3126602..3127160	expressed protein
LOC_Os09g06620	VII	chr09: 3139076..3140371	F-box domain containing protein
LOC_Os09g06660	VIII	chr09: 3163690..3164915	expressed protein
LOC_Os09g06670	IX	chr09: 3166948..3167453	expressed protein
LOC_Os09g06680	X	chr09: 3168154..3171995	G-beta repeat domain containing protein
LOC_Os09g06690	XI	chr09: 3180559..3180768	expressed protein
LOC_Os09g06719	XII	chr09: 3196606..3197534	expressed protein
LOC_Os09g06740	XIII	chr09: 3205933..3207190	zinc finger, C3HC4 type domain containing protein
LOC_Os09g06750	XIV	chr09: 3208362..3209057	expressed protein
LOC_Os09g06770	XV	chr09: 3236285.. 3237000	RING finger protein

Supplementary Table 2. Agronomic traits of *GSE9* knockout lines in the Zhonghua11 background.

Traits	ZH11	ZH11-Cas9-1	ZH11-Cas9-2
Plant height (cm)	97.44±1.85	97.65±1.46 (NS, p = 0.72)	96.88±1.77 (NS, p = 0.37)
Flag leaf length (cm)	26.54±2.05	26.45±2.40 (NS, p = 0.90)	26.58±2.63 (NS, p = 0.97)
Flag leaf width (cm)	1.49±0.05	1.49±0.05 (NS, p = 0.99)	1.50±0.04 (NS, p = 0.72)
Panicle numbers per plant	10.28±1.13	10.24±1.25 (NS, p = 0.92)	10.00±1.37 (NS, p = 0.52)
Panicle length (cm)	20.58±0.63	20.57±0.88 (NS, p = 0.98)	20.71±0.73 (NS, p = 0.56)
Primary branches per panicle	10.94±0.94	10.64±0.99 (NS, p = 0.37)	10.41±0.71 (NS, p = 0.07)
Secondary branches per panicle	35.00±2.33	35.88±1.69 (NS, p = 0.21)	35.71±1.69 (NS, p = 0.31)
Grain numbers per main panicle	159.61±19.04	158.29±15.18 (NS, p = 0.82)	159.71±15.13 (NS, p = 0.99)
Grain length (mm)	7.48±0.08	7.89±0.06 (**, $p = 1.00 \times 10^{-19}$)	7.82±0.07 (**, $p = 1.04 \times 10^{-15}$)
Grain width (mm)	3.36±0.04	3.28±0.03 (**, $p = 3.96 \times 10^{-8}$)	3.21±0.03 (**, $p = 1.97 \times 10^{-13}$)
Grain length/Grain width	2.23±0.05	2.41±0.02 (**, $p = 2.83 \times 10^{-21}$)	2.44±0.02 (**, $p = 1.40 \times 10^{-21}$)
Grain thickness (mm)	2.26±0.04	2.17±0.04 (**, $p = 2.53 \times 10^{-8}$)	2.17±0.03 (**, $p = 1.58 \times 10^{-9}$)
1,000-grain weight (g)	23.66±0.56	22.01±0.72 (**, $p = 3.86 \times 10^{-9}$)	21.95±0.61 (**, $p = 5.36 \times 10^{-10}$)
Grain yield per plant (g)	25.76±2.07	23.14±2.08 (**, $p = 4.88 \times 10^{-4}$)	22.35±2.08 (**, $p = 6.45 \times 10^{-6}$)
Seed setting rate per plant (%)	93.98±1.62	93.11±1.48 (NS, p = 0.11)	93.55±1.05 (NS, p = 0.37)

Note: Significant differences were determined by two-tailed Student's *t*-tests (NS, not significant; **, $p < 0.01$). Source data are provided as a Source Data file.

Supplementary Table 3. Agronomic traits of *GSE9* knockout lines in the Nipponbare background.

Traits	NIP	NIP-Cas9-1	NIP-Cas9-2
Plant height (cm)	75.05±1.72	75.47±2.35 (NS, p = 0.53)	75.35±2.14 (NS, p = 0.64)
Flag leaf length (cm)	24.66±1.84	24.37±1.99 (NS, p = 0.65)	25.03±1.59 (NS, p = 0.53)
Flag leaf width (cm)	1.38±0.08	1.39±0.08 (NS, p = 0.59)	1.41±0.07 (NS, p = 0.29)
Panicle numbers per plant	11.45±1.79	11.65±1.46 (NS, p = 0.72)	11.63±0.96 (NS, p = 0.73)
Panicle length (cm)	19.02±1.15	19.27±1.33 (NS, p = 0.54)	19.23±1.09 (NS, p = 0.57)
Primary branches per panicle	8.84±0.76	8.60±0.68 (NS, p = 0.30)	8.59±0.71 (NS, p = 0.31)
Secondary branches per panicle	19.26±1.41	20.05±2.61 (NS, p = 0.09)	19.88±1.36 (NS, p = 0.19)
Grain numbers per main panicle	121.00±5.23	120.55±5.71 (NS, p = 0.74)	119.59±8.02 (NS, p = 0.88)
Grain length (mm)	7.47±0.05	7.90±0.08 (**, $p = 8.01 \times 10^{-22}$)	7.72±0.10 (**, $p = 8.12\times 10^{-12}$)
Grain width (mm)	3.27±0.02	3.20±0.05 (**, $p = 6.56 \times 10^{-7}$)	3.20±0.04 (**, $p = 1.11 \times 10^{-7}$)
Grain length/Grain width	2.28±0.04	2.47±0.04 (**, $p = 4.94 \times 10^{-21}$)	2.42±0.05 (**, $p = 1.85 \times 10^{-13}$)
Grain thickness (mm)	2.24±0.02	2.21±0.02 (**, $p = 5.11 \times 10^{-4}$)	2.20±0.02 (**, $p = 6.83 \times 10^{-4}$)
1,000-grain weight (g)	24.40±0.62	22.91±0.75 (**, $p = 3.87 \times 10^{-8}$)	22.77±0.70 (**, $p = 5.39 \times 10^{-9}$)
Grain yield per plant (g)	24.86±1.56	22.70±1.26 (**, $p = 2.40 \times 10^{-5}$)	22.45±1.23 (**, $p = 7.39 \times 10^{-6}$)
Seed setting rate per plant (%)	89.67±1.92	89.50±2.38 (NS, p = 0.81)	89.89±1.77 (NS, p = 0.72)

Note: Significant differences were determined by two-tailed Student's *t*-tests (NS, not significant; **, $p < 0.01$). Source data are provided as a Source Data file.

Supplementary Table 4. Summary of *Ka* and *Ks* analysis of the reading frames between *Oryza* species with or without the start codon of *GSE9*.

**Species pair (*GSE9*/*gse9*)**	***Ka***	***Ks***	***Ka/Ks***
W3074 (Or-III)/W2302 (Or-I)	0.0131	0.0224	0.5861
W3074 (Or-III)/W2271 (Or-I)	0.0131	0.0111	1.1810
W3074 (Or-III)/*O. barthi*	0.0131	0.0224	0.5861
W3074 (Or-III)/*O. longistaminata*	0.0537	0.0826	0.6503
W3020 (Or-III)/W2302 (Or-I)	0.0131	0.0111	1.1810
W3020 (Or-III)/W2271 (Or-I)	0.0131	0.0224	0.5861
W3020 (Or-III)/*O. barthi*	0.0131	0.0111	1.1810
W3020 (Or-III)/*O. longistaminata*	0.0537	0.0702	0.7649

Note: Based on coding sequences of *GSE9* type and the orthologous non-coding sequence of *gse9* type. *Ka*, nucleotide divergence at nonsynonymous sites; *Ks*, nucleotide divergence at synonymous sites.

Supplementary Table 5. The statistical test for the estimated parameters of genetic differentiation between *japonica* and *indica* for *GSE9* locus and its flanking genomic regions.

**Parameters**	***GSE9* locus**	***GSE9* flanking regions**	***P* value**
Haplotype F_{ST}	0.36	0.12	1.80×10^{-11}
Nucleotide F_{ST}	0.86	0.71	3.03×10^{-5}
Nei's G_{ST}	0.22	0.07	2.91×10^{-12}
Hudson's G_{ST}	0.20	0.06	3.92×10^{-12}
Hudson's H_{ST}	0.20	0.06	3.93×10^{-12}

Note: Statistical analysis was performed by two-tailed one sample *t*-test.

Supplementary Table 6. The statistical test for the relative ratio of nucleotide diversity of cultivated rice to common wild rice between *GSE9* locus and its flanking genomic regions.

***π* ratio**	***GSE9* locus**	***GSE9* flanking regions**	***P* value**
Oryza sativa japonica/*Oryza rufipogon*	0.0558	0.1503	1.17×10^{-19}
Oryza sativa indica/*Oryza rufipogon*	0.7686	0.6381	1.00×10^{-10}

Note: Statistical analysis was performed by two-tailed one sample *t*-test.

Supplementary Table 7. Agronomic traits of *GSE9*-complementary lines in the Kasalath background.

Traits	Kasalath	Kasalath-C1	Kasalath-C2
Plant height (cm)	132.52±3.21	132.77±3.32 (NS, p = 0.83)	133.20±2.90 (NS, p = 0.54)
Panicle length (cm)	24.09±1.03	24.55±1.27 (NS, p = 0.24)	24.64±1.06 (NS, p = 0.13)
Number of productive tillers per plant	10.27±1.83	10.13±1.71 (NS, p = 0.83)	10.43±2.10 (NS, p = 0.83)
Flag leaf length (cm)	32.53±4.55	34.78±5.01 (NS, p = 0.17)	32.68±3.74 (NS, p = 0.92)
Flag leaf width (cm)	1.37±0.06	1.34±0.05 (NS, p = 0.12)	1.33±0.07 (NS, p = 0.19)
Primary branches per panicle	9.40±0.91	9.53±0.99 (NS, p = 0.70)	9.47±0.99 (NS, p = 0.85)
Secondary branches per panicle	34.40±4.97	34.73±6.01 (NS, p = 0.87)	33.47±4.72 (NS, p = 0.60)
Grain numbers per panicle	160.80±13.42	163.60±16.21 (NS, p = 0.61)	161.0±14.14 (NS, p = 0.97)
Grain length (mm)	7.62±0.20	7.47±0.27 (*, p =4.21 × 10^{-2})	7.44±0.24 (*, p = 1.33 × 10^{-2})
Grain width (mm)	2.33±0.20	2.48±0.22 (*, p = 1.37 × 10^{-2})	2.52±0.18 (**, p = 8.95 × 10^{-3})
Grain length/width ratio	3.31±0.27	3.11±0.27 (*, p = 1.57 × 10^{-2})	3.08±0.30 (**, p = 7.51 × 10^{-3})
1,000-grain weight (g)	15.93±0.90	15.91±0.74 (NS, p = 0.96)	15.89±1.05 (NS, p = 0.93)

Note: Significant differences were determined by two-tailed Student's *t*-tests (NS, not significant;**, $p < 0.01$; *, $p < 0.05$). Source data are provided as a Source Data file.
