# Supplementary material for: A de novo evolved gene contributes to rice grain shape difference between indica and japonica
Source: Nat Commun. 2023 Sep 22;14:5906. doi: 10.1038/s41467-023-41669-w (PMC10516980; doi:10.1038/s41467-023-41669-w)
Supplement: Supplementary file 3 — Description of Additional Supplementary Files [file 41467_2023_41669_MOESM3_ESM.pdf]

Description of Additional Supplementary files

Supplementary Data 1. List of 131 *indica* varieties and 158 *japonica* varieties used in GWAS analysis.

Supplementary Data 2. The expression levels of major regulators of grain shape in young panicle of the wild-type and *GSE9* transgenic plants using the RNA-seq data. Note: Different letters indicate statistically significant differences at $P < 0.05$ by one-way ANOVA test.

Supplementary Data 3. Information for accessions, database sources and start codon locus of *GSE9* in various *Oryza* groups.

Supplementary Data 4. List of primers used in this study.
