# Supplementary material for: A de novo evolved gene contributes to rice grain shape difference between indica and japonica
Source: Nat Commun. 2023 Sep 22;14:5906. doi: 10.1038/s41467-023-41669-w (PMC10516980; doi:10.1038/s41467-023-41669-w)
Supplement: Supplementary file 8 — Reporting Summary [file 41467_2023_41669_MOESM8_ESM.pdf]

nature portfolio

Corresponding author(s): Zefeng Yang, Aihong Li, Jinling Huang, Chenwu Xu

Last updated by author(s): Sep 6, 2023

Reporting Summary

Nature Portfolio wishes to improve the reproducibility of the work that we publish. This form provides structure for consistency and transparency in reporting. For further information on Nature Portfolio policies, see our Editorial Policies and the Editorial Policy Checklist.

Statistics

For all statistical analyses, confirm that the following items are present in the figure legend, table legend, main text, or Methods section.

n/a	Confirmed	
☐	☒	The exact sample size (*n*) for each experimental group/condition, given as a discrete number and unit of measurement
☐	☒	A statement on whether measurements were taken from distinct samples or whether the same sample was measured repeatedly
☐	☒	The statistical test(s) used AND whether they are one- or two-sided<br>*Only common tests should be described solely by name; describe more complex techniques in the Methods section.*
☒	☐	A description of all covariates tested
☐	☒	A description of any assumptions or corrections, such as tests of normality and adjustment for multiple comparisons
☐	☒	A full description of the statistical parameters including central tendency (e.g. means) or other basic estimates (e.g. regression coefficient) AND variation (e.g. standard deviation) or associated estimates of uncertainty (e.g. confidence intervals)
☐	☒	For null hypothesis testing, the test statistic (e.g. *F*, *t*, *r*) with confidence intervals, effect sizes, degrees of freedom and *P* value noted<br>*Give P values as exact values whenever suitable.*
☒	☐	For Bayesian analysis, information on the choice of priors and Markov chain Monte Carlo settings
☒	☐	For hierarchical and complex designs, identification of the appropriate level for tests and full reporting of outcomes
☒	☐	Estimates of effect sizes (e.g. Cohen's *d*, Pearson's *r*), indicating how they were calculated

Our web collection on statistics for biologists contains articles on many of the points above.

Software and code

Policy information about availability of computer code

Data collection

The SNP data of 289 rice natural population accessions, including japonica (PMID: 34615543) and new re-sequencing indica varieties, were used in this study.
The sequencing data for RNA-seq of 3 independent rice samples were generated from the Illumina NovaSeq 6000 system.
The untargeted metabolite profiling of 6 independent rice samples was performed using liquid chromatography-mass spectrometry (LC-MS) platform.

Data analysis

GWAS analysis:
A total of 2,269,131 SNPs were used for association analysis with a minor allele frequency (MAF) of > 0.01 and missing rate< 20 %. GWAS was performed based on the general linear model in tassel v5.2.4.
RNA-seq analysis:
The high-quality sequencing reads were mapped to the rice reference genome with HISAT2 v2.2.1. The read numbers mapped to each gene was calculated using featureCounts v2.0.1. The different expression gene analysis was analyzed by DESeq2 v1.36.0.
Metabolite profiling analysis:
Peak identification and metabolite identification were performed using the XCMS software v3.12.0. The Variable Importance for the Projection (VIP) was calculated by the orthogonal partial least squares discriminant analysis (OPLS-DA). VIP >1 and P value < 0.05 were used as the criteria to determine the differential metabolites.
Population genetic and evolutionary analyses:
The geographical information of cultivated groups was obtained from RFGB, and marked on map using Cartopy package v0.20.0 in Python v3.6.0 software.
The genetic difference parameters were estimated using PopGenome package v2.1.6 in R v4.1.2 software within 5-kb sliding windows between japonica and indica groups.

The average FST values in each 100-kb window were also estimated at the whole-genome level between indica and japonica subspecies using VCFtools v0.1.16.
The nucleotide diversity (π) and Neutral test (Tajima's D) of each population were calculated in 50-kb windows using VCFtools v0.1.16.
The phylogenetic tree was constructed using a maximum likelihood (ML) method by IQ-TREE v.2.1.2.
The haplotype network was calculated using pegas package v1.2 in R v4.1.2 software.

For manuscripts utilizing custom algorithms or software that are central to the research but not yet described in published literature, software must be made available to editors and reviewers. We strongly encourage code deposition in a community repository (e.g. GitHub). See the Nature Portfolio guidelines for submitting code & software for further information.

Data

Policy information about availability of data

All manuscripts must include a data availability statement. This statement should provide the following information, where applicable:

- Accession codes, unique identifiers, or web links for publicly available datasets
- A description of any restrictions on data availability
- For clinical datasets or third party data, please ensure that the statement adheres to our policy

Gene resequencing data are available in the European Variation Archive (EVA) at EMBL-EBI under accession number PRJEB65579 (https://www.ebi.ac.uk/eva/?eva-study=PRJEB65579).
RNA-seq data generated as part of the study have been deposited to the NCBI GEO database under the BioProject accession GSE218565 (https://www.ncbi.nlm.nih.gov/geo/query/acc.cgi?acc=GSE218565).
The full-length cDNA of GSE9 sequence confirmed by RACE is available through NCBI GenBank with accession number OR050540.1 (https://www.ncbi.nlm.nih.gov/nuccore/OR050540).
The genomic sequences of GSE9 in 1697 cultivated rice varieties were downloaded from the Rice Functional Genomics and Breeding Database (RFGB) (http://www.rmbreeding.cn/).
The genomic sequences of GSE9 in wild rice accessions were downloaded from OryzaGenome (http://viewer.shigen.info/oryzagenome2detail/index.xhtml) or Gramene (https://www.gramene.org/).
The A/G variations in GSE9 start codon site of 192 O. sativa, 19 O. rufipogon and 8 O. barthii accessions were obtained from the Rice Super Pan-genome Information Resource Database (RiceSuperPIRdb) (http://www.ricesuperpir.com/).
The DNA methylation levels in the genomic region of GSE9 were obtained from RiceENCODE (http://glab.hzau.edu.cn/RiceENCODE/).

Human research participants

Policy information about studies involving human research participants and Sex and Gender in Research.

Reporting on sex and gender	None.
Population characteristics	None.
Recruitment	None.
Ethics oversight	None. No human participants used in this research.

Note that full information on the approval of the study protocol must also be provided in the manuscript.

Field-specific reporting

Please select the one below that is the best fit for your research. If you are not sure, read the appropriate sections before making your selection.

☒ Life sciences ☐ Behavioural & social sciences ☐ Ecological, evolutionary & environmental sciences

For a reference copy of the document with all sections, see nature.com/documents/nr-reporting-summary-flat.pdf

Life sciences study design

All studies must disclose on these points even when the disclosure is negative.

Sample size

For the phenotype statistic for the transgenic lines, at least 15 samples were investigated in field for the students' t test analysis to determine the phenotype traits (PMID: 35189026).
For the Histological and cellular analysis, at least 5 samples were observed (PMID: 35189026).
For the expression level quantification of genes, 3 biological replicates were performed for each sample (PMID: 35189026).
For RNA-seq analysis, about 200 mg of young panicles were collected from the transgenic lines and wild-type plant, and 3 independent repeats of each sample (PMID: 35189026, 36307423).
For metabolite profiling analysis, metabolites were extracted from young panicles with six biological replicates (PMID: 30144334).
For the students' t test, at least 3 independent samples were required, so the number of samples were selected more than 3.
No statistical methods were used to predetermine sample sizes.
The sample size was determined according to the reports in the related research subjects, e.g., PMID: 35189026, 36307423, 30144334, etc.

Data exclusions	No data were excluded from our analysis.
Replication	At least 3 replicates were used for each experiment.
Randomization	All samples were arranged randomly into experimental groups.
Blinding	The experiments were used to compare the phenotype traits between transgenic lines and wild-type plants, therefore, binding is not relevant to this study.

Reporting for specific materials, systems and methods

We require information from authors about some types of materials, experimental systems and methods used in many studies. Here, indicate whether each material, system or method listed is relevant to your study. If you are not sure if a list item applies to your research, read the appropriate section before selecting a response.

Materials & experimental systems

n/a	Involved in the study
☐	☒ Antibodies
☒	☐ Eukaryotic cell lines
☒	☐ Palaeontology and archaeology
☒	☐ Animals and other organisms
☒	☐ Clinical data
☒	☐ Dual use research of concern

Methods

n/a	Involved in the study
☒	☐ ChIP-seq
☒	☐ Flow cytometry
☒	☐ MRI-based neuroimaging

Antibodies

Antibodies used	Anti-6x His antibody (Sigma-Aldrich, Cat. # SAB2702220, Clone No. GT161, dilution 1:8000)<br>Goat Anti-Mouse IgG-HPR secondary antibody (CWBIO, Cat. # CW0102S, dilution 1:10000)
Validation	The Anti-6x His antibody validation could be found in the website: https://www.sigmaaldrich.cn/CN/zh/product/sigma/sab2702220<br>The Goat Anti-Mouse IgG-HPR secondary antibody validation could be found in the website: https://www.cwbio.com/goods/index/id/10118
